# Extract of Green Walnut Shells, in Chronic Congestion of the Tonsils

**Published:** 1845-10

**Authors:** 


*Extract of green Walnut Shells, in Chronic Congestion of the Tonsils.* By Dr. Becker.—In the *Medicinishe Zeitung*, there is published, by Dr. Becker, a formula for a preparation, which he has employed successfuliy, in the case of a boy afflicted with congestion of the tonsils. The tumefaction had taken place many years previously, and was then so extensive, that speaking had become very difficult, and respiration often impossible during the night. Dr. Becker advised the following solution to be applied with a brush.—Extract of green walnut shells, 4 grammes; distilled waters, 60 grammes. To mix and dissolve according to art. This was so prompt in its effects, that the congestion of the tonsils had disappeared before the whole of the solution had been used.—*Journal de Medicine*, from *Lond. and Edin. Monthly Journal of Med. Science*, *March*, 1845.—*Ibid.*

